# Green Extraction Methods for Recovery of Antioxidant Compounds from Epicarp, Seed, and Seed Tegument of Avocado var. Hass (*Persea americana* Mill.)

**DOI:** 10.1155/2022/1965757

**Published:** 2022-07-04

**Authors:** Juan F. Grisales-Mejía, Harlen Torres-Castañeda, Margarita M. Andrade-Mahecha, Hugo A. Martínez-Correa

**Affiliations:** ^1^Facultad de Ingeniería y Administración, Departamento de Ingeniería, Universidad Nacional de Colombia-Sede Palmira, Carrera 32 # 12–, 00 Palmira, Valle del Cauca, Colombia; ^2^Facultad de Ingeniería y Administración, Departamento de Ciencias Básicas, Universidad Nacional de Colombia-Sede Palmira, Carrera 32 # 12–, 00 Palmira, Valle del Cauca, Colombia

## Abstract

The present study compared the extracts obtained from the epicarp, seed, and seed tegument of avocado var. Hass with pressurized liquid extraction (PLE) and ultrasound-assisted extraction (UAE). The extracts were quantified in terms of total phenolic content (TPC) and antioxidant capacity (AC). The PLE extracts had a global yield (*X*_0_) like that obtained with UAE using ethanol (Et) as the solvent. For the TPC, the extracts obtained with both techniques showed no significant differences (*p* > 0.05). On the other hand, the epicarp extracts obtained with PLE had higher values for AC: 829.8 *μ*mol TE/gDe (ABTS) and 3,215.1 *μ*mol Fe^2+^/g De (FRAP), recorded for UAE/Et. The AC in the avocado residue extracted with PLE suggested a high potential for applications in food, pharmaceutical, and cosmetology products.

## 1. Introduction

Avocado (*Persea americana* Mill.) is a subtropical fruit of the *Lauraceae* family originally from Central America, specifically from Guatemala and Mexico [1]. Today, its production has spread to countries such as the Dominican Republic, Peru, Colombia, Chile, Brazil, Kenya, and Indonesia, [2] which has made it possible to have more than 500 varieties available worldwide [3]. In this regard, the most commonly known avocado varieties are “Bacon,” “Fuerte,” “Reed,” “Choquette,” “Booth 8,” “Simmonds,” and “Hass,” the latter of which is the most commercially relevant one [3]. The Hass variety is characterized by being small, ovoid, and irregular in shape and having a rough epicarp that turns from green to purple as it matures [4]. It is usually consumed fresh in sandwiches, salads, and juices and due not only to the sensory properties of its pulp and the potential health benefits associated with its intake, but also to its content of mono- and polyunsaturated fatty acids, fiber, potassium, tocopherol, carotenoids, sterols, and other phytochemical compounds [5–7]. On an industrial level, its oil fraction has become very popular, so it is commonly found either directly in supermarkets or grocery stores or indirectly in cosmetic and food products [8]. Unfortunately, the amount of agroindustrial residues, mainly comprised of epicarp and seeds resulting from their use, is expected to increase considerably over time, taking into account that they represent approximately 30% of fresh fruits [9]. To reduce the amount of organic matter released into the environment from the use of avocado pulp, different researchers have extracted, analyzed, and characterized the bioactive compounds present in the epicarp and seeds, making it possible to demonstrate that they possess important antioxidant and antimicrobial properties [10–13]. While this is just a starting point for their potential use, the extraction techniques used for obtaining them could be limiting. Although they lead to achieve good results, these methods use volatile organic solvents (VOS) and require long operating times. Unfortunately, the toxicity of VOS generates negative impacts on the environment and limits the application of the extracts obtained for products for human consumption [14]. For this reason, to make a full contribution to the protection of the environment, the need has arisen to employ sustainable extraction processes. These methods, also known as green techniques, are characterized by the use of solvents that are generally recognized as safe (GRAS), which is relevant for the protection of the environment and in the application of the compounds obtained in products for human consumption [14]. In the case of avocado residues, nonconventional processes have been used to extract bioactive compounds, such as microwaves [15], ultrasound [13, 16, 17], supercritical fluids [18], and pressurized liquids [11, 12, 19]. Nevertheless, only a small number of studies have been carried out thus far.

The pressurized liquid extraction (PLE) process, also known as accelerated solvent extraction (ASE), is known to perform well at high pressures and temperatures [20, 21]. The operating pressures and temperatures allow fast penetration of the solvent into the solid matrix, by reducing its viscosity and surface tension and even modifying its polarity and dielectric constant, which favors mass transfer phenomena depending on the nature of the analyte to be extracted and thus improves extraction results in comparison to those of techniques conducted under atmospheric pressure conditions [22, 23]. Additionally, PLE is a very attractive process as it allows the use of GRAS solvents, such as ethanol and water, and has a high potential to be scalable on an industrial basis [14].

In this sense, the objective of this study was to compare the extracts obtained from the epicarp, the seed, and the seed tegument (seed husk) of the Hass avocado (*Persea americana* Mill.). The parameters investigated were as follows: (1) extraction technique, comprising PLE and assisted extraction by ultrasound (UAE); (2) type of solvents comprising 70% ethanol/water, 70% acetone/water, and ethanol, and (3) sample matrix: epicarp, seed, and seed tegument (husks).

## 2. Materials and Methods

### 2.1. Raw Material

The avocado (*Persea americana* Mill. var. Hass) was purchased from a supermarket (Cali, Colombia). Randomly selected fruits were stored at room temperature until the epicarp turned purple, indicating that the physiological maturity for consumption had been reached. The epicarp (*E*) was separated from the rest of the fruit manually, while the seed tegument (ST) was separated from the seed (*S*) using tweezers. These fractions were submerged in a sodium hypochlorite solution (100 ppm) for 5 min to prevent the appearance of microorganisms. Then, they were cut into homogeneous sizes and placed in a drying oven with forced circulation at 313 K, air flow velocity 3 m/s for 15 h [24], until reaching 10% moisture. Subsequently, the dried samples were reduced in size in a hammer mill until reach a particle size between 0.6 and 0.2 mm. The resulting samples were stored in individual containers with a light and oxygen barrier under refrigeration (277 K).

### 2.2. Chemicals

The following reagents were used in the extraction process: acetone (Merck, Germany), distilled water, and ethanol (Merck, Germany). For the characterization of the extracts, the following were used: hydrochloric acid (Sigma-Aldrich, Germany), glacial acetic acid (Fisher Chemical, USA), anhydrous sodium carbonate (Fisher Chemical, USA), potassium peroxydisulfate (ITW Reagents, Germany), 2,2-diphenyl-1-picrylhydrazyl (Sigma-Aldrich, Germany), 2,2′-azinobis (3-ethylbenzothiazolin-6-sulfonic acid) (Roche, Germany), 2,4,6–Tris (2-pyridyl)-s-triazine, TPTZ (Sigma-Aldrich, Germany), iron (III) chloride hexahydrate (Sigma-Aldrich, Germany), sodium acetate (Carlo Erba Reagent, Spain), gallic acid (Sigma-Aldrich, Germany), methanol (J.T. Baker, USA), Folin-Ciocalteu reagent (Merck, Germany), 6-hydroxy-2,5,7,8-tetramethylchroman-2-carboxylic acid, Trolox (Sigma-Aldrich, Germany), and Ferrous sulfate heptahydrate (Sigma-Aldrich, Germany). CO2 (purity 99.9%, v/v) was obtained from Cryogas (Cali, Colombia) and ethanol (J.T. Baker, USA).

### 2.3. Ultrasound-Assisted Extraction (UAE)

A bath ultrasonic extraction system (Branson 2510; 45 kHz) was employed, and acetone : water (Ac : Ag) (70 : 30% v/v), ethanol (Et), and ethanol : water (Et : Ag) (70 : 30% v/v) were used as solvents, following the methodology described by Castañeda et al. [25], with some modifications. 250 mg (wet base, wb) of each sample were weighed on an analytical balance (Ohaus PA-124C) and placed inside 2 mL tubes; 1.5 mL of solvent (3x) was added to each tube, which was placed in ultrasound for 10 min at 298 K and subsequently centrifuged (7000 rpm, 10 min) (Fisher Scientific Mini Centrifuge), separating the supernatants from the precipitate. The supernatants were collected from each sample. The extraction was done in duplicate in each case.

### 2.4. Pressurized Liquid Extraction (PLE)

#### 2.4.1. Extraction Process

**Figure 1:**
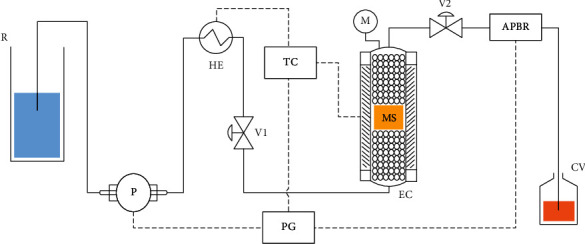
PLE unit scheme. R: solvent reservoir; P: solvent pump; HE: solvent heat exchanger; V1 and V2: blocking valves; EC: extraction cell; TC: temperature controller; M: manometer; ABPR: automatic back pressure regulator; CV: collecting vessel; PC: computer.

A high-pressure unit (SF100, Waters Co, Pittsburgh, USA) was used to carry out the extraction process.  Figure 1 shows the equipment scheme, which consists of a reservoir containing the solvent (R), solvent pump (P), blocking valves (V1 and V2), heat exchanger for the solvent (HE), stainless steel extraction cell (100 mL) with heating jacket (EC), temperature controller (TC), system pressure regulator (ABPR), and collection vessel (CV). The P, TC, and ABPR are controlled by a computer (PC). In each extraction, the EC bed was established as follows: 30 g of glass beads (GB), 40 g of GB + 1.5 g of matrix sample (wb), and 30 g of GB, respectively. The GBs disperse the solvent in the medium, preventing it from taking preferential paths and coming into close contact with the entire sample.

The PLE was carried out following the method used by Barrales et al. [26] and Figueroa et al. [19], with some modifications. Considering the results obtained from the extraction process described in Section 2.3, and its status as a *GRAS* solvent, Et was chosen as the extractive solvent. The PLE was developed in duplicate at 318 K, 225 bar, 180 min, and a flow rate of 5 g/min. These experimental conditions were chosen from a literature review, equipment limitations, and the gelatinization temperature of the starch present in the seed, which is approximately 346 K, according to Chel-Guerrero et al. [27].

#### 2.4.2. PLE Kinetic Experiments and Mathematical Modeling

For kinetic experiments (318 K, 225 bar), the extracts were collected in amber vessels every 5 min during the first 60 min and then every 20 min until 180 min. A total of 1.5 g (wb) of each matrix sample were used in each of the trial runs. The solvent was removed with rotary evaporation under reduced pressure (Heidolph, Hei-Vap Precision). The amber vessels with dry extract were stored under refrigeration (278 K) until further analysis.

In the extraction kinetics, the three different mass transfer mechanisms associated with the broken and intact cell (BIC) model and described by Sovová [28] were analyzed: constant extraction rate (CER), falling extraction rate (FER), and controlled diffusion (CD), in order to establish the influence of solvent flow on *X*_0_ and to set the total extraction time with maximum yield and minimum solvent consumption. The BIC model assumes that the analytes to be extracted have reached the cell surface thanks to grinding, which makes them more accessible to the solvent. Therefore, during the CER time, the mass transfer phenomenon known as convection predominates when obtaining all those analytes that are found on the surface of the cell. The FER time is considered as the transition period between the convection and diffusion phenomenon, which predominates in the DC period [29].

In this work, a spline model (empirical model), proposed by Meireles [30], was adapted from the data of the experiment obtained in each of the curves, using Microsoft Excel (v.16). The CER, FER, and CD periods were governed by the following equations:

For *t* ≤ *t*_CER_ (CER period),

(1) $$\begin{array}{c} y=a_{1}+k_{1}t. \end{array}$$

For *t*_CER_ ≤ *t* ≤ *t*_FER_ (FER period),

(2) $$\begin{array}{c} y=a_{1}+k_{1}t_{\text{CER}}+k_{2}\left(t-t_{\text{CER}}\right). \end{array}$$

For *t* ≥ *t*_FER_ (CD period),

(3) $$\begin{array}{c} y=a_{1}+k_{1}t_{\text{CER}}+k_{2}\left(t_{\text{FER}}-t_{\text{CER}}\right)+k_{3}\left(t-t_{\text{FER}}\right), \end{array}$$

where *y* is the accumulated mass (g) of the extract at the end of the period; *t* is the time (min); *t*_CER_ is the time length of CER (min); *t*_FER_ is the time length of FER (min); *a*_1_ is the linear coefficient of the CER period (g); *k*_1_, *k*_2_, and *k*_3_ are the slopes of the periods CER, FER, and CD (g.min^−1^), respectively [31].

#### 2.4.3. Global Extraction Yield (*X*_0_)

The global extraction yield (*X*_0_) of the extracts obtained with UAE and PLE was calculated with the following equation (Ec. 4):

(4) $$\begin{array}{c} X_{0}=\frac{D_{e}}{D_{w}}*100, \end{array}$$

where *D*_*e*_ is the weight of the dried extract and *D*_*w*_ (dry weight) is the weight of the dried sample of matrix (*E*, *S*, and ST).

### 2.5. Extracts Characterization

In the characterization of the extracts, the total phenolic content (TPC) and antioxidant capacity (AC) were considered using DPPH, ABTS, and FRAP. For this, concentrated solutions of 1 mL of methanol were made from the dried extracts obtained in each vessel from each of the samples. From these concentrated solutions, dissolutions were made with distilled water, which were considered standard samples (MP). Each characterization was done in triplicate.

#### 2.5.1. Estimation of Total Phenolic Content (TPC)

The TPC was evaluated using the Folin-Ciocalteu method [32], adapted to a 96-well microplate format according to the protocols established in the Chemistry Laboratory of the Universidad Nacional de Colombia–Palmira (Valle del Cauca, Colombia) [33]. From each MP, 60 *μ*L of sample was dispensed in each well of the microplate, followed by 60 *μ*L of the Folin-Ciocalteu reagent (RFC) and 180 *μ*L of sodium carbonate. The plate was incubated at 303 K for 30 min. Afterwards, reading was done at 750 nm in a microplate reader (Biotek ELx800). Standard dilutions of gallic acid with concentrations of 4 to 128 *μ*M were used to perform the calibration curve (*R*^2^ = 0.9905). The results were expressed as milligrams of gallic acid equivalent per gram (d.b.) of extract (mg GAE/g).

#### 2.5.2. Estimation of DPPH˙ Scavenging Capacity

The ability of the extracts to scavenging free radicals was measured following the procedure described by Castañeda et al. [25] and using the 2,2-diphenyl-1-picrylhydrazyl radical (DPPH). In each well of the microplate, 60 *μ*L of MP were mixed with 140 *μ*L of DPPH solution at 100 ppm. The microplates were incubated in the dark for 30 min at room temperature. The reading was done at 515 nm in the microplate reader. A calibration curve was prepared using Trolox as a standard at different concentrations (16-512 *μ*M) (*R*^2^ = 0.9756). The results were expressed as micromol of Trolox equivalent per gram of dry extract (*μ*mol TE/gDe).

#### 2.5.3. Estimation of ABTS^+^ Scavenging Capacity

The ability of the extracts to remove the radical cation ABTS^+^ was evaluated in accordance with Kong et al. [34] using the microplate format. ABTS^+^ (radical cation) was produced by mixing 38.4 mg of ABTS, 6.6 mg of potassium peroxodisulfate, and distilled water to a volume of 10 mL. The mixture was vigorously stirred and incubated at room temperature in the dark for 16 hours. This solution was diluted with distilled water (1 : 50) to obtain an absorbance of 0.900 at 750 nm. Then, 60 *μ*L of MP, at various concentrations, were mixed with 240 *μ*L of ABTS^+^ solution using the wells of a microplate. The absorbance decrease was measured at 750 nm after 1 minute of stirring. Trolox solutions were used as the standard for the elaboration of the calibration curve (4-128 *μ*M) (*R*^2^ = 0.9932). The results were reported as micromol of Trolox equivalent per gram of dry extract (*μ*mol TE/gDe).

#### 2.5.4. Estimation of FRAP (Ferric Reducing Antioxidant Power)

The measurement of the ability of the extracts to reduce ferric ions (Fe^3+^) to ferrous ions (Fe^2+^) was taken in accordance with the procedure described by Arancibia-Avila et al. [35], with some variations. FRAP reagent was prepared by mixing aqueous solutions of 300 mM acetate buffer (pH 3.6), 10 mM TPTZ in 40 mM HCl, and 20 mM ferric chloride hexahydrate in at a 10 : 1 : 1 v/v/v ratio. For the assay, 140 *μ*L of the freshly prepared FRAP reagent was mixed with 60 *μ*L of MP using the wells of a microplate. After 30-min incubation at room temperature, the microplate was read at 630 nm. Aqueous solutions (32-1024 *μ*M) of ferrous sulfate heptahydrate were used to elaborate the calibration curve (*R*^2^ = 0.9756). The results were expressed as micromol of Fe^2+^ equivalent per gram of dry extract (*μ*mol Fe^2+^/gDe).

### 2.6. Statistical Analysis

A completely randomized design (extraction method) was used to study the effect of extraction technique (UAE and PLE), solvent (acetone : water, ethanol, and ethanol : water), and (3) sample matrix (epicarp, seed, and seed tegument) on response variables (X0, TPC, DPPH, ABTS, and FRAP). Statistical analyzes were carried out using MATLAB® (R2021a) and Microsoft Excel (v16.0). A one-way ANOVA and a post hoc Tukey test (*p* < 0.05) were used to verify significant differences. The results were expressed as the mean ± standard deviation (SD). All experiments were performed in triplicate (*n* = 3).

## 3. Results and Discussion

### 3.1. Global Extraction Yield

When analyzing the influence of solvents used in UAE on the *X*_0_ in each sample (Table 1), it was found that the Ac : Ag mixture had the highest values for the epicarp (13.2%) and seed (21.4%). These results did not show significant differences (*p* > 0.05) from those obtained with Et : Ag in the same fractions: 12.4% and 19.7%, respectively. Meanwhile, the *X*_0_ of the ethanol extracts of the epicarp (9.3%) and seed (9.7%) was significantly different. On the other hand, the seed tegument (ST), the epicarp (*E*), and seed (*S*) obtained the best *X*_0_ with Ac : Ag, while the results for Et and Et : Ag did not show significant differences (*p* > 0.05). In general, as a trend, it was observed that the *S* extracts had the highest *X*_0_ with all the solvents, followed by the *E* and (ST) although, in some cases, there were no significant differences between the samples. The higher yield obtained from *S* using aqueous mixtures was probably due to the presence of soluble fibers in this part of the fruit. It is known that the avocado var. Hass seed is rich in starch and soluble fiber [29, 36]. Although in most cases where bioactive compounds are obtained, the best yields and qualities of the metabolites are obtained with solvents that are not considered GRAS, and their application is limited, especially in products for human consumption [14].

The PLE technique obtained an *X*_0_ of 11.9%, 11.2%, and 9.5% in *S*, *E*, and ST, respectively, showing the same trend as UAE/Et. The *X*_0_ of the E and S extracts did not show significant differences (*p* > 0.05). Although the *X*_0_ was not similar to that obtained with the UAE/Ac : Ag mixture, an increase was observed with respect to UAE/Et for each sample, probably as a result of the operation variables set in the PLE. Machado et al. [37] reported an increase in the *X*_0_ (12.10%, 14.27%, and 14.99%) when acidified water, used for obtaining bioactive compounds from *Rubus fruticosus* L. This the same trend was obtained by Santos et al. [38] in the extract of *Chrysopogon zizanioides* roots, as the temperature was increased (313, 323, and 333 K) using any of the extraction solvents (i.e. ethanol, ethyl acetate, or hexane). Figueroa et al. [19] evaluated the incidence Et : Ag ratio on the *X*_0_ and phenolic compounds extracted from Hass avocado epicarp. The authors found that the best *X*_0_ (39%) was presented at 473 K and an Et : Ag ratio of 1 : 1 (v/v), at 110 bar. This result is higher than that reported in the present investigation for the same matrix (11.1%), may be associated with the high temperature, which can favor the extraction not only of antioxidant compounds, but also and organic acids, soluble sugars, and fiber. High pressure (225 bar) and polarity of ethanol in PLE technique causes break the interactions metabolites–matrix, such as the Van der Waals, hydrogen, and dipole-dipole molecular bonds this results in enhanced mass transfer rate [20]. Additionally, the nature of the matrix plays an important role, due to the difference in the amount of extractable analytes, which is evident in the behavior of the extraction yield: *S* > *E* > ST.

### 3.2. Extract Characterization

#### 3.2.1. Total Phenolic Content (TPC)

**Figure 2:**
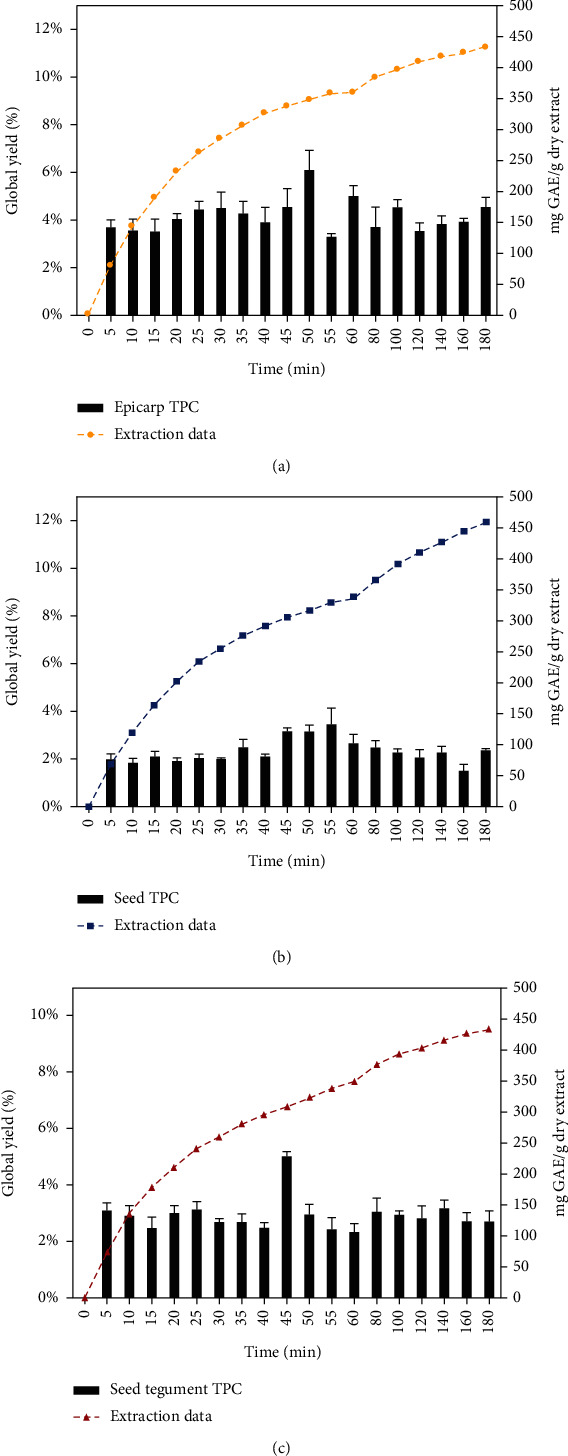
Kinetics of extraction, TPC, and global yield by PLE: (a) epicarp, (b) seed, and (c) seed tegument of Hass avocado.

When analyzing the TPC in the UAE extracts, the significant impact of the solvents used was evident. The highest phenolic content was found in the Ac : Ag extract, with *E* extract having the highest value (208.5 mg GAE/g) (Figure 2). The results obtained from the ST with A c: Ag (167 mg GAE/g) and Et (110 mg GAE/g) do not show significant differences (Table 1). This behavior was also observed in the study conducted by Rodríguez-Carpena et al. [39]. These authors analyzed the differences presented in *E* extracts, pulp, and *S* of two varieties of avocado: Fuerte and Hass, obtained with solid-liquid extraction at room temperature with ethyl acetate and 70% acetone and methanol solutions as solvents. The results were conclusive in establishing that acetone extracts, and especially the *E* of the two varieties had the highest TPC values (899.7 mg GAE/g dry matter). In other case, Tremocoldi et al. [16] quantified the TPC in avocado var. Fuerte and Hass extracts using an Et : Ag mixture (80 : 20 v/v) with the UAE method. The results in the *E* and *S* were lower than those reported in the present study; 63.5 and 57.3 mg GAE/g of lyophilized sample. Several factors could be related to this difference: the geographical origin of avocados is not the same, so the climate and soil conditions where they were grown could affect, and the maturity of the fruit and the difference in the in the extractive solvent. Wang et al. [9] found that the TPC of *S* extracts obtained with UAE using a solution of acetone/water/acetic acid (70 : 29.7 : 0.3, v/v/v) of different varieties, including avocado var. Hass (51.6 mg GAE/g fresh sample; FS), was higher than that found in *E* extracts (12.6 mg GAE/gFS).

On the other hand, the PLE seed extract had the lowest TPC (90.1 mg GAE/g De). The best results were found in the *E* (158.8 mg GAE/g), followed by the ST (132.8 mg GAE/g De), maintaining the behavior recorded in all UAE extracts (Figure 2). The TPC values in the PLE extracts were lower than those of UAE/Et, despite having exhibited an increase in *X*_0_. This is because the PLE process was carried out at fixed temperature (318 K) and pressure values (225 bar), which were probably not the best for obtaining a high TPC, but possibly they did make it possible to obtain other compounds. In the work of Figueroa et al. [19], a TPC value of 88 mg GAE/g was reported for an PLE extract of *E*, obtained at 473 K and 110 bar, using Et : Ag (1 : 1 v/v) as solvent. In this case, the authors associated the results with the decrease in the value of the solvent's dielectric constant, which was 26 at that temperature. Therefore, among the reasons that can be associated with the difference compared to what was obtained in the present study (158.8 mg GAE/g) are the solvent (pure ethanol) and extraction temperature (318 K). Other reasons could be subject to the conditions in which the culture was developed and the maturation stage at the time of characterization, as previously mentioned.

#### 3.2.2. Antioxidant Capacity (AC)

The main function of an antioxidant compound is to retard the oxidation of other molecules by inhibiting chain reactions made by free radicals, thus reducing oxidative damage [40]. Three methods were used to evaluate the AC of extracts obtained from different parts of the avocado: DPPH, ABTS^+^, and FRAP.

In DPPH, the AC values ranged from 1009 *μ*mol TE/g to 2651.9 *μ*mol TE/g for the PLE/Et/S and UAE/Et/S, respectively. As is known, the higher the value, the better AC of the extract. So, in relative terms, the UAE/Et : Ag/ST, UAE/Et/S, PLE/Et/E and UAE/Et/E extracts (1754.5, 1348, 1241.8, and 1227.5 *μ*mol TE/g, respectively) had a moderate AC. Among the three samples studied, the extract of the fraction obtained with PLE that presented a higher AC with the DPPH and FRAP method was the epicarp, with 1329.4 *μ*mol TE/g and 3251.1 *μ*mol Fe^2+^/g, respectively. On the other hand, the ST (931.5 *μ*mol TE/g) obtained the highest value with the ABTS cation technique. With the UAE, secondary metabolites of ST obtained with Et and Et : Ag had the highest quantifications in the three spectrophotometric techniques (Table 1). The solvents used under this method (UAE) only registered significant differences (*p* < 0.05) in the ST. These differences can be attributed to the affinity of the solvent or mixture of solvents with the analytes of interest present in this plant matrix. In general terms, extracts obtained with UAE had better values than those obtained with PLE in both TPC and DPPH, while PLE extracts showed better values in the spectrophotometric measurements, corresponding to the ABTS and FRAP techniques, except for the extract with UAE/Et/TS, which had the highest AC in FRAP (3,900.9 *μ*mol Fe^2+^/g).

**Figure 3:**
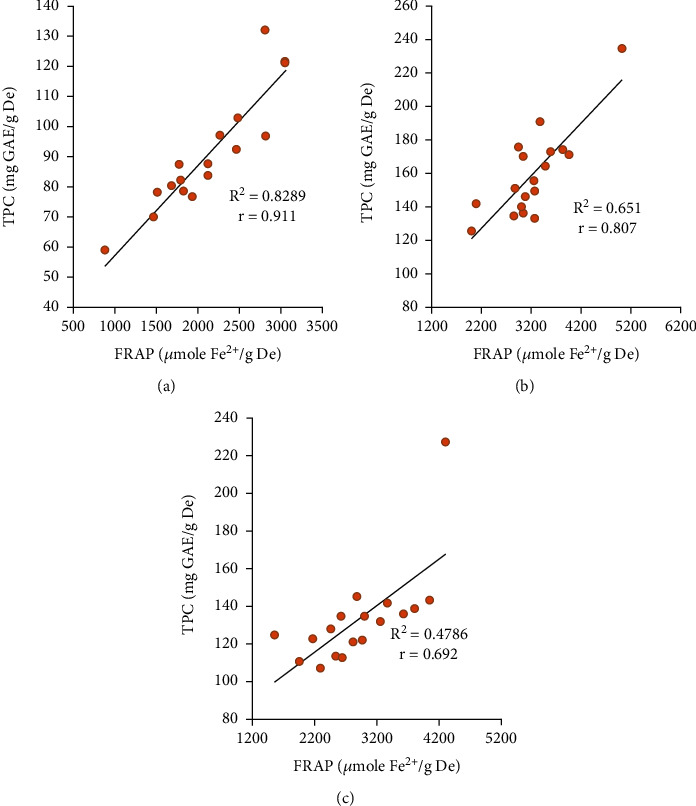
Correlation between TPC and antioxidant capacity (FRAP) of extracts of (a) seed, (b) epicarp, and (c) seed tegument.

The FRAP method measures the reduction of the ferric ion (Fe^3+^) to the ferrous ion (Fe^2+^) caused by an antioxidant, forming complexes with TPTZ and generating an intense blue coloration.  Figure 3 shows the Pearson correlation coefficient (*r*) between the TPC and AC (FRAP) data obtained in the samples of interest. All correlations were greater than 0 (*r* > 0), highlighting the value obtained for the *S* (*r* = 0.911) and *E* (*r* = 0.807), which were close to 1. This confirms that the antioxidant capacity of the fractions is related to the TPC.

Various results can be found in the literature regarding the antioxidant property of avocado var. Hass residues [16, 41]. Kosińska et al. [42] evaluated the AC of epicarp and seed extracts of avocado Hass obtained with conventional extraction (thermostatic bath) at 333 K with a methanol solution (80%) for 15 min. The phenolic compounds of the avocado var. Hass epicarp (25.32 mg catechin equivalent/g) exhibited the highest values with ABTS (0.161 *μ*mol TE/g). Authors have associated epicarp AC with the presence of procyanidin dimers and catechins. On the other hand, Tremocoldi et al. [16] found that for avocado *var.* Fuerte residues, the values were higher than those of *var.* Hass with the DPPH method; the seeds of both varieties had high values. However, the ABTS and FRAP readings of the epicarp were above those of the seeds. The ABTS results for the avocado *var.* Hass epicarp of that study had a value (791.5 *μ*mol TE/g) close to that obtained from extracts of the same fraction, extracted using PLE (829.8 *μ*mol TE/g) in the present study.

For the FRAP determination, the PLE results (3,215.1 *μ*mol Fe^2+^/g and 2114.4 *μ*mol Fe^2+^/g) were much higher than that reported by Tremocoldi et al. [16] (1175.1 *μ*mol Fe^2+^/g and 656.9 *μ*mol Fe^2+^/g) for the epicarp and seed, respectively. In this study, the ABTS values for the seed and seed tegument obtained with PLE were superior to those found by Figueroa et al. [12] (432 and 300 *μ*mol TE/g, respectively). The differences in the behavior of antioxidant capacities recorded by the different single electron transfer techniques (SET) may be due to the interaction of the different radicals with the metabolites present in the extracts, such as the origin of the raw material in relation to the biotic and abiotic factors to which it was exposed. The high antioxidant capacities of avocado var. Hass residues could be associated with the wide variety of families of compounds they contain [42]. López-Cobo et al. [13] recorded the presence of quercetin derivatives: quercetin-diglucoside, quercetin-3-O-arabinosyl-glucoside, and rutin, in addition to chlorogenic acid, perseitol, and quinic acid in the epicarp. In addition, in the seed, to perseitol, quinic acid, and citric acid, hydroxytyrosol-1-glycoside, tyrosol hexoside, and hydroxycinnamic acids were found. In these residues, it has also been found organic acids, hydroxycinnamic acids, hydroxybenzoic acids, phenolic alcohol derivatives, flavonoids such as luteolin-7-O-(2^″^-O-pentosyl) hexoside and quercetin, catechin monomers, dimers, trimers, and tetracyl procyanidins, among other polar compounds [11, 12].

### 3.3. Mathematical Modeling

The parameters of the spline model can be seen in Table 2. The parameter *k*_1_ of all the residual matrices is greater than the parameters *k*_2_ and *k*_3_. The parameter *k*_1_ corresponds to the extraction rate or slope (g.min^−1^), of the analytes during the CER period (*t*_CER_). In this period, the mass transfer is governed by convection, which means that the extraction solvent extracts all those analytes that are easily removed as they are on the surface of the particle. For this reason, it has been established that during the CER period, it is possible to achieve between 50% and 75% of the amount of total extract obtained during the process [29]. In this sense, at the end of *t*_CER_ (Table 2), a percentage of 51.9% for *E*, 50.9% for *S*, and 53.1% for ST was estimate. Regarding *k*_2_, this parameter refers to the extraction rate of the analytes during the FER period (*t*_FER_). The decrease in its value with respect to *k*_1_ is because this period is considered as a transition period between a fast extraction rate (CER) and a slow one (DC), in which the mass transfer mechanisms that act are convection and diffusion, respectively. Therefore, during the FER period, the combination of these two mechanisms occurs: on the one hand, convection decreases when the metabolites present on the surface of the particle are depleted, and on the other hand, diffusion begins, transporting the metabolites from the inside the particle to the outside, being the main reason for the decrease in the rate [25]. Usually, at the end of the FER period, 75%-90% of the overall extraction yield can be achieved. In this case, the percentages of extracts obtained in *t*_FER_ (Table 2) of the total amount reported at the end of the process for *E*, *S*, and TS were 80.9%, 77%, and 85%, respectively. As can be seen in Table 2, the time at which the FER period ends (*t*_FER_) for each matrix is less than 70 min, which is much shorter than the total extraction time (180 min). This means a decrease in operational costs (energy, solvents, labor, etc.), considering that to extract between 15% and 20% of the missing extract. It would be necessary to spend approximately 120 min, which corresponds to the DC period.

**Figure 4:**
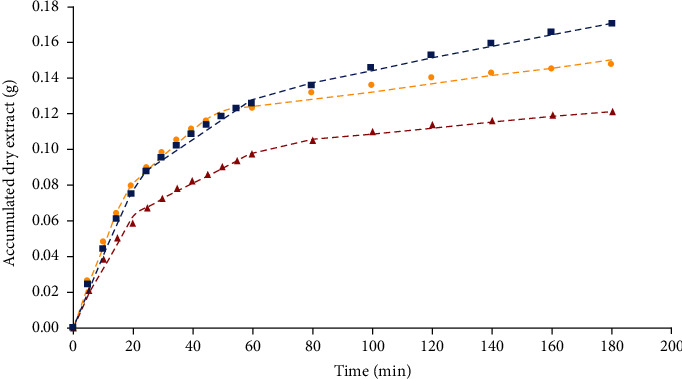
Spline mathematical model fitted to the experimental data of epicarp (circle), seed (square), and seed tegument (triangle). The line “dot, dot, dot” is the model fitted to epicarp, “dash, dot, dash” for seed, and “dash, dash, dash” for seed tegument.

Once the DC period has been reached, the extraction rate slows down, which characterizes the low values of *k*_3_ (g.min^−1^). All these phenomena can be seen in Figure 4.

This information (Table 2) is useful to design and scale up the processes. By knowing the extraction time (*t*_CER_ or *t*_FER_) and the solvent mass flow (g.min^−1^) when it enters the system, the amount of solvent used in the process can be calculated. Once this amount is known, the solvent-feed ratio can be found, also known as S/F (g/g), at which the desired yield is obtained. Therefore, when scaling the operation, it is desirable to maintain the same S/F value and thus be able to calculate both the solvent flow and the necessary extraction time, depending on the amount of feed to be used. However, it is important that prior to scaling the process, the operating parameters to be worked on are previously optimized. Once the scaling prediction is made, the economic feasibility of the process can be analyzed by estimating the cost of manufacturing (COM) [29].

To increase *X*_0_ and obtaining components that are still in the biological matrix after extraction, innovative processes are being carried out, making use of the difference in polarity that solvents can have and the extraction properties that they acquire at certain pressures and temperatures, depending on the method applied [41]. These processes are known as sequential extractions or biorefinery processes, managing supercritical fluid extraction.

(SFE), liquids expanded by means of a compressed gas (GXL), and PLE, among others. In these sequential processes, the use of mathematical modeling of each stage is relevant to be able to establish the efficiency of the global process.

## 4. Conclusion

According to the total content of phenols and the antioxidant capacity of the extracts obtained from the processing residues of Hass avocado (epicarp, seed, and seed tegument), these matrices could be a potential source of compounds of high value for different industries. The recovery of extracts with antioxidant functionality from theses wastes using green techniques such as ultrasound-assisted extraction and pressurized liquid extraction could decrease negative impacts on environmental, by means of the use GRAS solvents. On the other hand, extraction with pressurized fluids is a technique that would make it possible to apply the notions of circular economy as it is considered a green process and thus be an alternative to the need to implement more sustainable processes that allow contributing to the fulfillment of some Sustainable Development Goals. In this sense, the results for avocado var. Hass residues extracted with PLE suggest high potential for applications in food, pharmaceutical and cosmetology products.

## Figures and Tables

**Table 1:** Global yield (*X*_0_), total phenolic content (TPC), and antioxidant capacity (AC) of extracts obtained by UAE and PLE.

Method	Solvent	Sample	*X*_0_ (%)	TPC^1^	AC		
					DPPH^2^	ABTS^2^	FRAP^3^
EAU	Ac : Ag	E	13.2 ± 0.8^b^	208.5 ± 19.8^a^	1,183.1 ± 11.1^bc^	355.9 ± 0.6^a^	2,312.0 ± 33.8^bcd^
		S	21.4 ± 0.7^a^	110.6 ± 8.8^def^	1,135.8 ± 18.8^bc^	353.4 ± 4.8^a^	2,213.9 ± 38.3^bcd^
		ST	12.9 ± 0.9^b^	167.2 ± 8.9^abcd^	1,167.3 ± 37.4^bc^	350.4 ± 12.4^a^	2,282.3 ± 32.7^bcd^
	Et	E	9.3 ± 0.3^c^	183.4 ± 6.0^abc^	1,227.5 ± 42.2^bc^	358.3 ± 13.4^a^	2,284.6 ± 79.3^bcd^
		S	9.7 ± 0.2^c^	110.9 ± 3.3^def^	1,348.0 ± 96.4^bc^	343.3 ± 25.0^a^	2,074.0 ± 53.8^cd^
		ST	5.5 ± 0.2^d^	161.9 ± 13.5^abcd^	2,651.9 ± 38.0^a^	650.9 ± 47.6^a^	3,900.9 ± 70.3^a^
	Et : Ag	E	12.4 ± 0.6^b^	192.6 ± 11.1^ab^	1,138.70 ± 72.7^bc^	339.9 ± 22.3^a^	2,101.8 ± 46.9^cd^
		S	19.7 ± 1.0^a^	94.5 ± 4.5^ef^	1,081.2 ± 48.9^bc^	332.3 ± 15.1^a^	2,033.4 ± 57.9^cd^
		ST	4.5 ± 1.0^d^	133.3 ± 9.4^bcdef^	1,754.5 ± 58.3^b^	475.4 ± 38.0^a^	2,920.2 ± 75.8^abcd^
PLE	Et	E	11.2 ± 0.07^bc^	158.8 ± 25.9^abcd^	1,329.4 ± 492.1^bc^	829.8 ± 445.4^a^	3,215.1 ± 668.4^ab^
		S	11.9 ± 0.05^bc^	90.1 ± 19.02^f^	977.1 ± 212.9^c^	572.1 ± 206.5^a^	2,114.4 ± 587.6^d^
		ST	9.5 ± 0.16^c^	132.8 ± 26.0^cde^	1,283.0 ± 319.2^bc^	931.5 ± 346.6^a^	2,908.6 ± 730.6^abc^

*E*: epicarp; *S*: seed; ST: seed tegument. ^1^mg GAE/g de; ^2^*μ*mol ET/g de; ^3^*μ*mol Fe^2+^/g de. Equal letters in the same columns indicate that there are no significant differences (*p* < 0.05). Values were expressed as mean ± SD.

**Table 2:** Parameters of the model by parts obtained from the PLE extraction kinetics of epicarp (*E*), seed (*S*), and seed tegument (ST) of avocado var. Hass at 318 K, 225 bar and Et as solvent.

Parameters	*E*	*S*	ST
*a*_1_ (g)	2.63 × 10^−3^	3.86 × 10^−3^	4.28 × 10^−3^
*k*_1_ (g.min^−1^)	4.32 × 10^−3^	3.73 × 10^−3^	2.95 × 10^−3^
*t*_CER_ (min)	17.45	22.41	20.52
*y*_CER_	0.0780	0.0875	0.0648
*k*_2_ (g.min^−1^)	1.44 × 10^−3^	1.08 × 10^−3^	8.6 × 10^−4^
*t*_FER_ (min)	47.73	64.16	66.23
*y*_FER_	0.1216	0.1323	0.1040
*k*_3_ (g.min^−1^)	2.2 × 10^−4^	3.4 × 10^−4^	1.6 × 10^−4^
*R* ^2^	0.9755	0.9858	0.9803

## Data Availability

The data that supports the findings of this study are available upon request.
